# Anti-TNF antibody therapy induces IL-17 suppressing regulatory T cells in patients with rheumatoid arthritis

**DOI:** 10.1186/ar3597

**Published:** 2012-02-09

**Authors:** Jenny McGovern, Clare A Notley, Dao Nguyen, Claudia Mauri, David A Isenberg, Michael R Ehrenstein

**Affiliations:** 1 Division of Medicine, University College London, London WC1E 6JF, UK

## 

Biologic therapies not only offer the prospect of improved patient outcomes in a variety of autoimmune diseases, but also the opportunity to explore the specific target's role in the underlying mechanisms of disease. Over recent years we have studied the role of regulatory T cells (Treg) in patients with rheumatoid arthritis before and after anti-TNF therapy. We have shown that Treg from patients with rheumatoid arthritis have defective suppressor function. This Treg defect is linked with abnormalities in the expression and function of CTLA-4. Anti-TNF antibody therapy did not reverse CTLA-4 dysfunction but instead induced the differentiation of a distinct and potent Treg population. These induced Treg were able to inhibit IL-17 production, in contrast to Treg from healthy individuals, patients with active RA or RA patients treated with etanercept, a modified TNF receptor. These results may provide mechanistic insight into the therapeutic benefit of switching between different anti-TNF agents and the differing incidence of tuberculosis between adalimumab and etanercept.

